# Transcranial Direct Current Stimulation in Patients with Prolonged Disorders of Consciousness: Combined Behavioral and Event-Related Potential Evidence

**DOI:** 10.3389/fneur.2017.00620

**Published:** 2017-11-21

**Authors:** Ye Zhang, Weiqun Song, Jubao Du, Su Huo, Guixiang Shan, Ran Li

**Affiliations:** ^1^Department of Rehabilitation Medicine, Xuan Wu Hospital, Capital Medical University, Beijing, China

**Keywords:** transcranial direct current stimulation, coma recovery scale-revised, event-related potentials, P300, disorders of consciousness

## Abstract

**Background:**

The electrophysiological evidence supporting the therapeutic efficacy of multiple transcranial direct current stimulation (tDCS) sessions on consciousness improvement in patients with prolonged disorders of consciousness (DOCs) has not been firmly established.

**Objectives:**

To assess the effects of repeated tDCS in patients with prolonged DOCs by Coma Recovery Scale-Revised (CRS-R) score and event-related potential (ERP).

**Method:**

Using a sham-controlled randomized double-blind design, 26 patients were randomly assigned to either a real [five vegetative state (VS) and eight minimally conscious state (MCS) patients] or sham (six VS and seven MCS patients) stimulation group. The patients in the real stimulation group underwent 20 anodal tDCS sessions of the left dorsolateral prefrontal cortex (DLPFC) over 10 consecutive working days. The CRS-R score and P300 amplitude and latency in a hierarchical cognitive assessment were recorded to evaluate the consciousness level before tDCS and immediately after the 20 sessions.

**Results:**

The intra-group CRS-R analysis revealed a clinically significant improvement in the MCS patients in the real stimulation group. The inter-group CRS-R analysis showed a significant difference in CRS-R between VS and MCS patients at baseline in both the real and sham stimulation groups. The intra-group ERP analysis revealed a significant increase in P300 amplitude after tDCS in the MCS patients in the real stimulation group, but no significant differences in P300 latency. For the inter-group ERP analysis, we observed significant differences regarding the presence of P300 at baseline between the VS and MCS patients in both groups.

**Conclusion:**

The repeated anodal tDCS of the left DLPFC could produce clinically significant improvements in MCS patients. The observed tDCS-related consciousness improvements might be related to improvements in attention resource allocation (reflected by the P300 amplitude). The findings support the use of tDCS in clinical practice and ERP might serve as an efficient electrophysiological assessment tool in patients with DOCs.

## Introduction

Patients with severe brain injury may suffer from a wide spectrum of prolonged disorders of consciousness (DOCs), mainly including vegetative state (VS) (1) and minimally conscious state (MCS) (2). Although, some studies have attempted to explore pharmacologic and non-pharmacologic treatment effects, there are currently no evidence-based guidelines on DOCs treatment.

Transcranial direct current stimulation (tDCS) is a form of non-invasive neurostimulation that involves modulating cortical excitability using weak polarizing currents. The dorsolateral prefrontal cortex (DLPFC) is one of the key brain regions of top-down control that has been shown to adjust the course of bottom-up processing through increases in extrastriate neural activity, thereby enhancing attention to stimuli (3, 4). Several studies have reported that tDCS of the left DLPFC can transiently improve working memory and cognitive control for emotional information in healthy participants (5–7). Other studies have demonstrated that a single session and multiple sessions of tDCS of the left DLPFC can transiently improve Coma Recovery Scale-Revised (CRS-R) total scores in MCS patients (8–10). Recently, a study used transcranial magnetic stimulation–electroencephalography (TMS-EEG) to assess the electrophysiological effects of tDCS (11). It was found that a single session of tDCS could modulate cortical excitability in patients with DOCs by stimulating the left DLPFC. However, the electrophysiological evidence supporting the therapeutic efficacy of multiple tDCS sessions on consciousness improvement in patients with prolonged DOCs has not been firmly established.

Currently, the most widely accepted tool for assessing consciousness is a behavioral scale. However, because of severe sensory and motor deficits, the lack of behavioral responsiveness of command following is not necessarily indicative of a lack of consciousness. It has been reported that behavioral abilities can fluctuate over time, which would lead to a high rate of misdiagnosis (12). Recently, event-related potential (ERP) recording has been used as an objective and easy evaluation method for assessing cortical information processing capabilities in the absence of overt behavior in patients with DOCs. Some studies have attempted to specifically detect the existence of attentional capabilities by measuring the P300 wave, which is well understood to be a correlate of attention and conscious perception (13–15).

The P300 amplitude mainly depends on stimulus saliency, and it reflects the level of attentional resource allocation (i.e., attentional load), and the P300 latency reflects the speed at which the target stimulus is detected and evaluated (i.e., task complexity) (16–18). In some patients with DOCs, the subject’s own name (SON) spoken by a familiar voice (which is an important self-related stimulus) can elicit a stronger cognitive response and activate the cerebral cortex more extensively than non-self-referential emotional stimuli (15, 19). Our previous study of patients with DOCs involved using the SON (spoken by a familiar voice) as the deviant stimulus, and a 1,000 Hz tone and the subject’s derived name (SDN) as the standard stimuli, and we successfully obtained a hierarchical auditory ERP pattern, indicating that the approach could be used as a valuable tool to evaluate patients with DOCs (15, 20, 21).

Given the significance of ERP to the assessment of electrophysiological changes related to consciousness recovery, we sought to explore ERP and behavioral evidence of consciousness recovery after anodal tDCS of the left DLPFC in VS and MCS patients.

## Materials and Methods

### Experimental Design

The study involved a sham-controlled randomized double-blind design, and all enrolled patients were randomly assigned to one of two groups: a real or sham stimulation group. In the real stimulation group, 20 tDCS sessions were administered over 10 consecutive working days (from Monday to Friday) and in the sham stimulation group, 20 sham stimulation sessions were administered over 10 consecutive working days (from Monday to Friday). All the eligible patients underwent behavioral and electrophysiological evaluation by blinded assessors at two time points: (1) baseline (pre-tDCS) and (2) immediately after the 20 active or sham sessions (post-tDCS).

### Patients

Twenty-six patients with severe brain injury (five VS and eight MCS patients in the real stimulation group, and six VS and seven MCS patients in the sham stimulation group) were recruited from the Department of Rehabilitation Medicine at Xuan Wu Hospital, Beijing, China, between July 2015 and June 2017. All the patients were right handed. In the real stimulation group, the etiologies of the patients’ conditions were anoxia (*n* = 2), traumatic brain injury (*n* = 5), hemorrhagic stroke (*n* = 5), and ischemic stroke (*n* = 1). In the sham stimulation group, the etiologies were anoxia (*n* = 3), traumatic brain injury (*n* = 7), hemorrhagic stroke (*n* = 2), and ischemic stroke (*n* = 1). The duration of VS or MCS ranged from 1.0 to 17.4 months (mean 5.7 ± 4.6 months) in the real stimulation group and 1.4 to 14.6 months (mean 5.0 ± 3.8 months) in the sham stimulation group. The general conditions of each of the included patients are shown in Table 1. None of them had a history of neurological disease prior to their coma. All the patients underwent at least one brain computed tomography (CT) or magnetic resonance imaging (MRI) scan. Four patients (VS4, MCS1, MCS2, and MCS5) in the real stimulation group and three (VS4, MCS1, and MCS2) in the sham stimulation group had undergone a left craniotomy to reduce intracranial pressure. None of the patients underwent craniotomy-related plastic surgery. None of them carried a metallic cerebral implant, pacemaker, or neurostimulator. Any other treatments or drugs which modify cortical excitability were excluded. During the 10 days tDCS, all patients received the basic rehabilitation program involving physiotherapy, speech therapy, as well as medical demands during the hospital stay. Written informed consents were acquired from all the patients’ families or caregivers. The study was conducted according to the Declaration of Helsinki, and the ethics approval was provided by the ethics committee of the hospital.

**Table 1:** Description of general conditions of the included DOCs.

Patients	Gender	Age	Months post-injury	Etiology	CT/MRI findings	Patients	Gender	Age	Months post-injury	Etiology	CT/MRI findings
	
Real stimulation						Sham stimulation					
VS1	M	49	5.6	Anoxic	Diffuse cortical and subcortical atrophy	VS1	M	48	3.1	Anoxic	Diffuse cortical and subcortical atrophy
VS2	F	27	17.4	Anoxic	Diffuse cortical and subcortical atrophy	VS2	M	47	2.3	Hemorrhagic	Bilateral parietal and temporal lesions
VS3	M	50	4.0	Hemorrhagic	Bilateral parietal and temporal lesions, diffuse cortical atrophy	VS3	F	26	8.2	Anoxic	Diffuse cortical and subcortical atrophy
VS4	F	73	9.2	Traumatic	Left parietal and temporal lesions	VS4	M	7	4.5	Traumatic	Left temporal lesions
VS5	F	39	2.4	Hemorrhagic	Bilateral pontine lesions	VS5	M	34	9.2	Traumatic	Left parietal lesions
MCS1	M	67	2.0	Traumatic	Bilateral frontal lesions	VS6	F	70	2.0	Traumatic	Left parietal and temporal lesions
MCS2	F	74	7.3	Traumatic	Left hemisphere lesion, diffuse cortical atrophy	MCS1	F	71	3.2	Traumatic	Left parietal lesions
MCS3	F	56	9.7	Traumatic	Diffuse white matter damage	MCS2	M	66	1.6	Traumatic	Bilateral frontal lesions
MCS4	M	56	7.0	Hemorrhagic	Right temporal lesions	MCS3	M	63	1.4	Hemorrhagic	Right frontal and temporal lesions
MCS5	F	72	4.8	Traumatic	Left temporal lesions	MCS4	F	64	4.5	Ischemic	Left thalamus-capsular lesion
MCS6	M	85	1.0	Ischemic	Left frontal and parietal lesions, diffuse atrophy	MCS5	M	9	3.4	Anoxic	Diffuse cortical and subcortical atrophy
MCS7	M	60	1.0	Hemorrhagic	Right parietal and occipital lesions	MCS6	M	35	14.6	Traumatic	Left parietal lesions
MCS8	M	53	2.3	Hemorrhagic	Right thalamus-capsular lesion	MCS7	F	69	5.6	Traumatic	Diffuse white matter damage

*CT, computed tomography; DOCs, disorders of consciousness; MRI, magnetic resonance imaging; VS, vegetative state; MCS, minimally conscious state; M, male; F, female*.

### tDCS Protocol

The tDCS was administered using an Eldith DC-stimulator (neuroConn GmbH, Ilmenau, Germany). A direct current was applied by the battery-driven constant-current stimulator using saline-soaked surface sponge electrodes (7 cm × 5 cm) with the anode placed over the left DLPFC (position F3 of the 10–20 international electroencephalography system for EEG placement) and the reference cathode placed over the right supraorbital region (approximately position Fp2). The stimulation parameters were slightly modified based on the previous studies (9, 10, 22). During the real tDCS, the current was increased to 2 mA from the onset of stimulation. Stimulation lasted 20 min per session, and it was administered twice a day (one session in the morning and one session in the afternoon) for 10 consecutive working days (from Monday to Friday). For the sham tDCS, the same electrode arrangement and stimulation parameters were employed, except that the stimulator was turned off after 30 s. For the patients who had undergone left craniotomy surgery (VS4, MCS1, MCS2, and MCS5 in the real stimulation group and VS4, MCS1, and MCS2 in the sham stimulation group), the current was increased to 1 mA instead of 2 mA from the onset of stimulation.

### Behavioral Assessment

After admission, the patients with DOCs were allowed to familiarize themselves with the ward environment for 3–4 days, and their clinical conditions were allowed to stabilize. Following 1 week of careful daily observation, the diagnoses were made by multiple trained and experienced blinded examiners using the CRS-R. The CRS-R is a sensitive tool for characterizing the level of consciousness and monitoring neurobehavioral recovery in patients with DOCs. In our study, each patient was assessed by the same two blinded assessors before and after tDCS. They performed separate assessments and then reached a consensus. To ensure patients’ best vigilance state, the clinical evaluations were performed at patients’ bed in the morning after routine nursing procedures.

### ERP Assessment

#### Procedure

We used two oddball paradigms, both of which have been described previously (15). In the first (Tone-SON paradigm, also called the TO paradigm), a 1,000-Hz tone was used as the standard stimulus, and in the second (SDN-SON paradigm, also called the DO paradigm), the SDN was used as the standard stimulus. SON was used as the deviant stimulus in both paradigms. The 1,000-Hz tone lasted for 100 ms and was generated using Adobe Audition software (Adobe, Beijing Fistar Technology Ltd. Co., Beijing, China). The SDN was formed by reversing the constituent order of the SON. For each patient, the two-character SON and SDN were recorded by a first-degree family member and digitized for binaural replay (at a maximum sound pressure level of 90 dB) during the experiment. The mean durations of the SON and SDN for the VS and MCS patients in the real and sham stimulation groups were not significantly different.

The patients were told that they were going to be played a series of sounds and that they were only required to listen. The stimuli were delivered in a random order, with an interstimulus interval of 0.8–1.2 s. Each paradigm consisted of 500 stimuli, and the probability of the deviant stimuli being administered was 0.2. The order of presentation of the two paradigms was balanced in all four groups of patients. The stimuli were presented using E-prime 2.0 software (Carnegie-Mellon University and University of Pittsburgh, Pittsburgh, PA, USA). The two paradigms lasted approximately 30 min in total.

#### ERP Recording

If the patient’s medical stability was adequate, the ERPs were recorded in the ERP experiment room, and if not, the recordings were carried out at the patient’s bedside. Patients cleaned their hair before test to decrease the electric resistance of the scalp. ERPs were recorded from midline electrodes Fz, Cz, and Pz with 64-channel electrode caps (Neuroscan Inc., Charlotte, NC, USA) according to the international 10–20 system. The reference electrode was placed on the nose, and the ground electrode on the mid forehead. An electrooculogram was acquired using two vertical electrodes placed above and below the left eye, and two horizontal electrodes placed 10 mm from the lateral canthi of the eyes. The electrode impedance was kept below 5 kΩ throughout the experiment. The bandpass was 0.1–100 Hz, and the sampling rate was 1,000 Hz. The ERP recording protocol was according to criteria previously published (15). It was performed while the patients were in a wakeful state, with eyes open, and with minimal ambient noise. To ensure the stability of the auditory information processing in the patients with DOCs (18), we recollected the ERP for those with initially low-quality ERP recordings, which potentially reduced false-negative ERP findings.

#### ERP Analysis

Electrooculogram artifacts were corrected using the method proposed by Semlitsch et al. (23). Each EEG was split into segments from 100 ms prestimulus to 700 ms poststimulus. Then, baseline adjustment was done to ensure that all ERP segments had the same origin. The EEG segment contaminated by amplifier clipping, bursts of electromyography activity, or peak-to-peak deflection exceeding ±100 µV were excluded from averaging. The EEG segments were averaged separately for deviant and standard stimuli. The ERP analysis protocol was according to criteria previously published (15). The recognition of P300 waves was performed by trained and experienced blinded assessors. Peak detection was used to obtain the P300 amplitudes and latencies. The P300 amplitude and latency in the patients with DOCs were calculated manually one by one.

### Statistical Analysis

We considered two different types of data: first the behavioral test scores (CRS-R) and second the ERP data (peak P300 latencies from stimulus onset and the P300 amplitudes from the baseline of the stimulus onset). Statistical analysis was performed on the averaged traces from each participant using SPSS version 22.0 (SPSS, Chicago, IL, USA).

Regarding the behavioral data, a repeated measure ANOVA with *Stimulation* (real, sham) × *Group* (VS, MCS) × *Time* (pre-tDCS, post-tDCS) mixed ANOVA was performed on CRS-R total scores and CRS-R subscale scores. For the CRS-R total scores at baseline, a two-way ANOVA with *Stimulation* (real, sham) × *Group* (VS, MCS) was performed.

Regarding the ERP data, a repeated measure ANOVA with *Stimulation* (real, sham) × *Paradigms* (TO, DO) × *Time* (pre-tDCS, post-tDCS) mixed ANOVA was performed on P300 amplitude and latency. A test for *post hoc* comparisons was used when the results reached significance at *p* < 0.05. Multiple comparisons using Bonferroni correction (*n* comparisons) had to be performed, and results were considered significant at *p* < (0.05/n). Fisher’s exact test was used to analyze dichotomous variables indicating the presence or absence of the P300 wave and patient diagnosis (VS or MCS) at baseline. The significance level was set at *p* = 0.05. All the data were analyzed using SPSS software.

## Results

### Patients’ Characteristics

The real and sham stimulation groups did not differ for important demographics (etiology, age, and time since onset). No adverse effects that were potentially related to tDCS were observed in any of the patients.

### CRS-R Results

In the real and sham stimulation groups, we compared the CRS-R total scores and CRS-R subscale scores at baseline (pre-tDCS) and after 20 sessions of tDCS (post-tDCS) in each group (VS and MCS), and the CRS-R total scores between each group (VS and MCS) at baseline.

**Figure 1:**
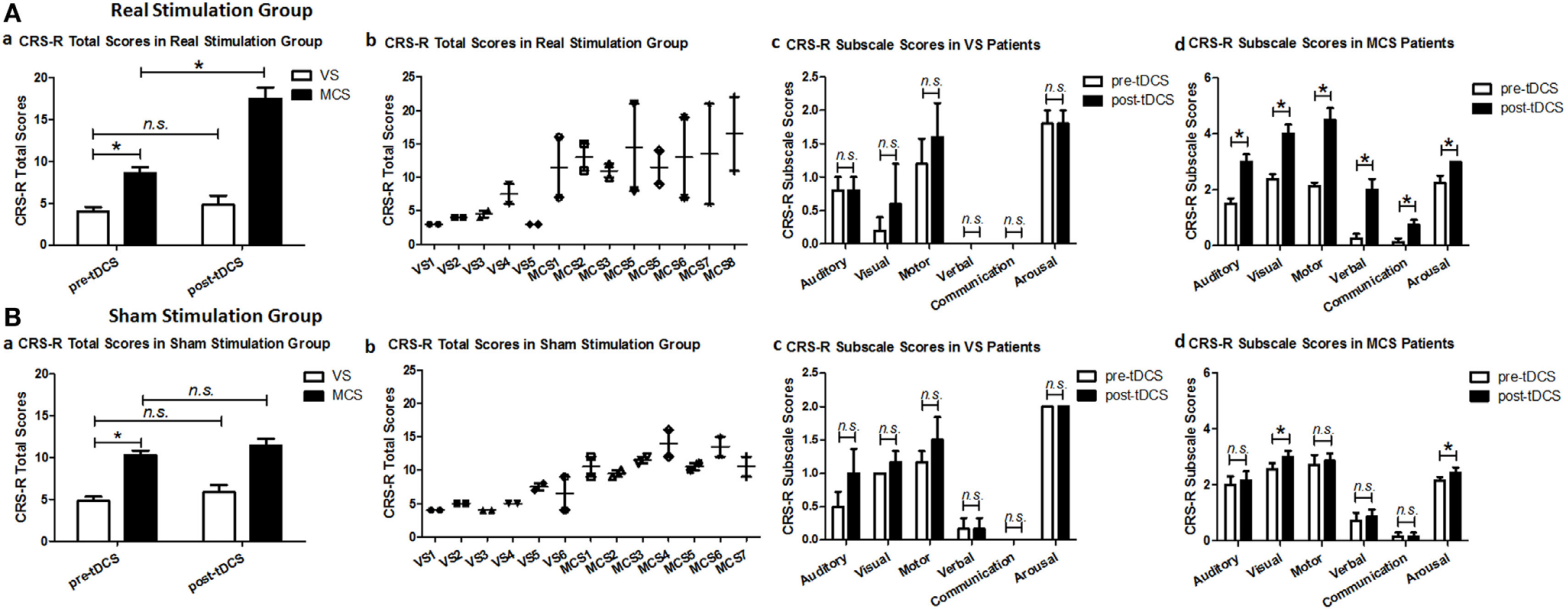
Coma Recovery Scale-Revised (CRS-R) total scores and CRS-R subscale scores for the vegetative state (VS) and minimally conscious state (MCS) patients in **(A)** the real stimulation group and **(B)** the sham stimulation group at baseline (pre-tDCS) and after 20 sessions of transcranial direct current stimulation (tDCS) (post-tDCS). *p < 0.05. Non-significant (n.s.): difference was not statistically significant.

Regarding the CRS-R total scores, the *Stimulation* (real, sham) × *Group* (VS, MCS) × *Time* (pre-tDCS, post-tDCS) repeated measures ANOVA revealed a main effect of *Time* [*F*(1, 22) = 30.48, *p* < 0.0005], a main effect of *Group* [*F*(1, 22) = 118.05, *p* < 0.0005], an interaction between *Time* and *Group* [*F*(1, 22) = 15.90, *p* = 0.001], an interaction between *Time* and *Stimulation* [*F*(1, 22) = 7.41, *p* = 0.012], and, most important, a significant three-way interaction [*F*(1, 22) = 8.36, *p* = 0.008]. *Post hoc* comparison revealed that after tDCS, a significant improvement was observed in the MCS patients in the real stimulation group [*F*(1, 22) = 72.54, *p* < 0.0005], but no significant difference was observed in the VS patients in the real stimulation group or in the sham stimulation group (*F* < 1), nor MCS patients in the sham stimulation group [*F*(1, 22) = 4.21, *p* = 0.052]. The two-way ANOVA analysis at baseline showed a significant main effect of *Group* [*F*(1, 22) = 70.23, *p* < 0.0005], and no significant main effect of *Stimulation* [*F*(1, 22) = 4.30, *p* = 0.05] or interaction between *Group* and *Stimulation* (*F* < 1), suggesting a significant difference in CRS-R total scores between the VS and MCS patients at baseline, as shown in Table 2 and Figure 1.

**Table 2:** CRS-R total scores and CRS-R subscale scores in VS and MCS groups at baseline (pre-tDCS) and after 20 sessions of tDCS (post-tDCS).

Real stimulation	CRS-R Scores		Sham stimulation	CRS-R scores	
	Pre-tDCS	Post-tDCS		Pre-tDCS	Post-tDCS
VS1	3 (1-0-0-0-0-2)	3 (1-0-0-0-0-2)	VS1	4 (0-1-1-0-0-1)	4 (0-1-1-0-0-2)
VS2	4 (1-0-1-0-0-2)	4 (1-0-1-0-0-2)	VS2	5 (1-1-1-0-0-2)	5 (1-1-1-0-0-2)
VS3	4 (1-0-1-0-0-2)	5 (1-0-2-0-0-2)	VS3	4 (0-1-1-0-0-2)	4 (0-1-1-0-0-2)
VS4	6 (1-1-2-0-0-2)	9 (1-3-3-0-0-2)	VS4	5 (1-1-1-0-0-2)	5 (1-1-1-0-0-2)
VS5	3 (0-0-2-0-0-1)	3 (0-0-2-0-0-1)	VS5	7 (1-1-2-1-0-2)	8 (2-1-2-1-0-2)
MCS1	7 (1-2-2-0-0-2)	16 (3-4-4-1-1-3)	VS6	4 (0-1-1-0-0-2)	9 (2-2-3-0-0-2)
MCS2	11 (2-3-3-0-0-3)	15 (3-3-4-1-1-3)	MCS1	9 (2-2-3-0-0-2)	12 (2-3-3-1-0-3)
MCS3	10 (2-3-2-0-0-3)	12 (2-3-3-1-0-3)	MCS2	9 (1-2-3-1-0-2)	10 (1-3-3-1-0-2)
MCS4	8 (1-2-2-1-0-2)	21 (4-5-5-3-1-3)	MCS3	11 (3-3-2-0-1-2)	12 (3-3-3-0-1-2)
MCS5	9 (2-3-2-0-0-2)	14 (2-3-3-1-0-3)	MCS4	12 (3-3-4-0-0-2)	16 (4-4-4-1-0-3)
MCS6	7 (1-2-2-0-0-2)	19 (3-4-5-3-1-3)	MCS5	10 (2-3-1-1-0-3)	11 (2-3-2-1-0-3)
MCS7	6 (1-2-2-0-0-1)	21 (3-5-6-3-1-3)	MCS6	12 (2-3-3-2-0-2)	15 (3-4-3-2-0-3)
MCS8	11 (2-2-2-1-1-3)	22 (4-5-6-3-1-3)	MCS7	9 (1-2-3-1-0-2)	12 (2-3-4-1-0-2)

*VS, vegetative state; MCS, minimally conscious state; CRS-R, Coma Recovery Scale-Revised; tDCS, transcranial direct current stimulation*.

For the CRS-R subscale scores, the repeated measures ANOVA and *post hoc* comparison revealed a significant improvement in the auditory [*F*(1, 22) = 29.97, *p* < 0.0005], visual [*F*(1, 24) = 22.96, *p* < 0.0005], motor [*F*(1, 22) = 54.78, *p* < 0.0005], verbal [*F*(1, 22) = 77.79, *p* < 0.0005], communication [*F*(1, 22) = 36.67, *p* < 0.0005], and arousal [*F*(1, 24) = 19.91, *p* < 0.0005] subscale scores in the MCS patients in the real stimulation group, but no significant difference was observed in the VS patients in the real stimulation group or in the sham stimulation group, as shown in Table 2 and Figure 1.

### ERP Results

In the real and sham stimulation groups, we compared the average latencies and amplitudes of P300 obtained using the midline electrodes (Fz, CZ, and Pz) at baseline (pre-tDCS) and after 20 sessions of tDCS (post-tDCS) in each group (VS and MCS), and the presence or absence of P300 between each group (VS and MCS) at baseline.

In our study, only one patient’s ERP (VS2 in sham stimulation group) was recorded at the patient’s bedside (both pre-tDCS and post-tDCS) instead of the ERP experiment room.

**Figure 2:**
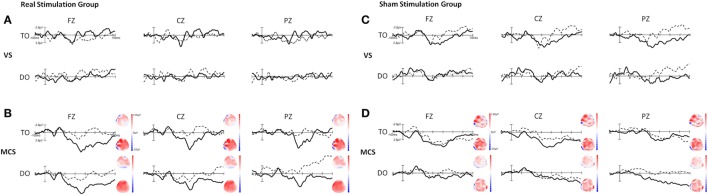
Grand averages of P300 waves in the VS and minimally conscious state (MCS) patients at Fz, Cz, and Pz for the TO and DO paradigms in **(A,B)** the real stimulation group and **(C,D)** the sham stimulation group. The dotted line represents the deviant stimuli [subject’s own name (SON)] at baseline (pre-tDCS) and the thick line represents the deviant stimuli (SON) after 20 sessions of transcranial direct current stimulation (tDCS) (post-tDCS). The right-side panels illustrate the P300 topographies at baseline (upper panel) and after 20 sessions of tDCS (lower panel) in the MCS patients. Blue indicates negative amplitude and red indicates positive amplitude.

We have reported that a P300 wave was present in both TO and DO paradigms in 16 healthy subjects in a previous study (Figure S1 in Supplementary Material) (20). Figure 2 shows the grand averages of P300 at baseline and after tDCS in the VS and MCS patients at Fz, Cz, and Pz for the two paradigms in the real and sham stimulation groups. In the real and sham stimulation groups, there were no P300 waves in either paradigm before or after tDCS in the VS patients. The P300 topographies in the MCS patients showed that the two paradigms elicited a more pronounced frontal P300 effect (both at baseline and after tDCS) in the real and sham stimulation groups.

**Figure 3:**
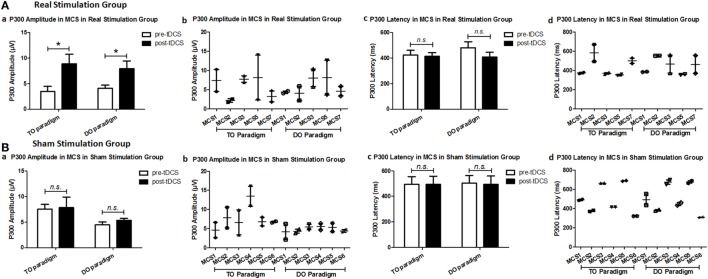
Average P300 amplitude and latency in the minimally conscious state (MCS) patients [**(A)** MCS1, MCS2, MCS3, MCS5 and MCS7 in the real stimulation group and **(B)** MCS1, MCS2, MCS3, MCS4, MCS5 and MCS6 in the sham stimulation group] for the TO and DO paradigms at baseline [pre-transcranial direct current stimulation (tDCS)] and after 20 sessions of tDCS (post-tDCS). *P < 0.05. Non-significant (n.s.): difference was not statistically significant.

Regarding the P300 amplitudes, the *Stimulation* (real, sham) × *Paradigms* (TO, DO) × *Time* (pre-tDCS, post-tDCS) repeated measures ANOVA revealed a main effect of *Time* [*F*(1, 18) = 9.25, *p* = 0.007], and an interaction between *Time* and *Stimulation* [*F*(1, 18) = 5.38, *p* = 0.032]. *Post hoc* comparison revealed that a significant increase in P300 amplitudes was observed in the MCS patients in the real stimulation group [*F*(1, 20) = 14.55, *p* = 0.001], but no significant difference was observed in the sham stimulation group (*F* < 1). Regarding the P300 latencies, a three-way repeated ANOVA revealed no significant main effect of *Time* [*F*(1, 18) = 4.26, *p* = 0.054], *Stimulation* (*F* < 1), or Paradigms (*F* < 1). The results are shown in Tables 3 and 4 and Figure 3. From the inter-group analysis of ERP values at baseline, we observed significant differences in the presence of P300 waves for the TO paradigm (χ^2^ = 5.923, *p* = 0.032) and the DO paradigm (χ^2^ = 6.964, *p* = 0.021) between the VS and MCS patients in the real stimulation group, as well as for the TO paradigm (χ^2^ = 6.741, *p* = 0.021) and the DO paradigm (χ^2^ = 6.198, *p* = 0.029) in the sham stimulation group according to Fisher’s exact test (Table 5).

**Table 3:** Amplitude of P300 (μV) in VS and MCS groups in the TO and DO paradigms at baseline (pre-tDCS) and after 20 sessions of tDCS (post-tDCS).

Real stimulation	P300 amplitude in TO paradigm						P300 amplitude in DO paradigms						Sham stimulation	P300 amplitude in TO paradigm						P300 amplitude in DO paradigms					
	Pre-tDCS			Post-tDCS			Pre-tDCS			Post-tDCS				Pre-tDCS			Post-tDCS			Pre-tDCS			Post-tDCS		
	Fz	Cz	Pz	Fz	Cz	Pz	Fz	Cz	Pz	Fz	Cz	Pz		Fz	Cz	Pz	Fz	Cz	Pz	Fz	Cz	Pz	Fz	Cz	Pz
VS1	–	–	–	–	–	–	–	–	–	–	–	–	VS1	–	–	–	–	–	–	–	–	–	–	–	–
VS2	–	–	–	–	-	–	–	–	–	–	–	–	VS2	–	–	–	–	–	–	–	–	–	–	–	–
VS3	–	–	–	–	–	–	–	–	–	2.40	4.75	4.40	VS3	–	–	–	–	–	–	–	–	–	–	–	–
VS4	–	–	–	10.45	4.62	1.70	–	–	–	8.11	4.10	1.05	VS4	1.30	2.89	4.81	3.93	4.01	2.48	12.05	11.27	4.69	2.69	4.20	3.72
VS5	3.73	3.38	3.71	0.45	0.88	2.15	–	–	–	1.16	1.69	2.20	VS5	8.85	9.14	0.47	7.01	7.47	7.03	–	–	–	–	–	–
MCS1	5.10	5.81	2.58	11.47	12.46	6.95	5.93	5.82	0.34	4.64	5.71	3.76	VS6	–	–	–	4.02	4.08	3.17	–	–	–	–	–	–
MCS2	0.71	2.01	2.45	2.14	3.05	2.66	2.10	3.59	0.85	4.41	7.40	5.82	MCS1	11.42	6.12	2.33	5.49	2.12	0.24	2.10	3.59	0.85	7.53	6.57	4.68
MCS3	8.00	7.10	5.60	5.86	8.13	12.00	6.13	6.67	4.58	10.77	10.91	9.52	MCS2	5.68	6.64	3.30	11.71	14.02	6.00	5.42	6.14	0	4.77	5.31	4.43
MCS4	4.03	3.47	1.98	3.73	4.25	3.76	–	–	–	4.25	8.36	3.72	MCS3	8.68	10.61	10.44	0.83	6.56	2.70	3.45	5.85	4.90	2.19	7.17	9.04
MCS5	6.53	0.37	0	19.90	19.11	3.07	5.96	3.33	1.59	22.45	10.20	5.30	MCS4	5.91	11.59	15.06	9.87	17.98	20.29	1.14	5.30	7.53	3.85	6.44	8.75
MCS6	–	–	–	4.21	2.25	1.80	1.32	3.67	4.70	7.03	5.24	4.84	MCS5	0.83	3.36	13.00	0.64	9.66	13.61	0	11.00	8.35	2.76	4.81	5.19
MCS7	0	0.08	5.43	6.53	7.99	12.36	0.70	4.18	5.27	4.38	5.95	7.31	MCS6	10.33	6.63	3.82	10.42	7.12	2.12	3.99	5.11	5.32	4.25	2.46	5.28
MCS8	10.73	11.32	19.62	4.75	5.26	4.06	–	–	–	4.21	4.31	4.91	MCS7	8.45	2.75	1.14	8.26	1.71	0.54	–	–	–	8.68	8.23	7.88

*VS, vegetative state; MCS, minimally conscious state; TO, Tone-SON; DO, SDN-SON; SON, subject’s own name; SDN, subject’s derived name; tDCS, transcranial direct current stimulation*.

**Table 4:** Latency of P300 (ms) in VS and MCS groups in the TO and DO paradigms at baseline (pre-tDCS) and after 20 sessions of tDCS (post-tDCS).

Real Stimulation	P300 latency in TO paradigm						P300 latency in DO paradigms						Sham stimulation	P300 latency in TO paradigm						P300 latency in DO paradigms					
	Pre-tDCS			Post-tDCS			Pre-tDCS			Post-tDCS				Pre-tDCS			Post-tDCS			Pre-tDCS			Post-tDCS		
	Fz	Cz	Pz	Fz	Cz	Pz	Fz	Cz	Pz	Fz	Cz	Pz		Fz	Cz	Pz	Fz	Cz	Pz	Fz	Cz	Pz	Fz	Cz	Pz
VS1	–	–	–	–	–	–	–	–	–	–	–	–	VS1	–	–	–	–	–	–	–	–	–	–	–	–
VS2	–	–	–	–	–	–	–	–	–	–	–	–	VS2	–	–	–	–	–	–	–	–	–	–	–	–
VS3	–	–	–	–	–	–	–	–	–	459	460	460	VS3	–	–	–	–	–	–	–	–	–	–	–	–
VS4	–	–	–	400	400	419	–	–	–	539	561	540	VS4	320	365	365	368	367	367	406	407	408	410	437	437
VS5	414	415	416	499	500	503	–	–	–	428	432	453	VS5	311	328	328	341	341	337	–	–	–	–	–	–
MCS1	407	369	367	368	365	365	385	383	382	411	376	376	VS6	–	–	–	588	587	510	–	–	–	–	–	–
MCS2	672	671	672	500	493	490	570	543	546	539	562	563	MCS1	464	505	484	493	498	507	570	543	547	451	430	428
MCS3	369	367	346	412	363	361	578	574	530	373	372	371	MCS2	405	372	370	371	369	369	384	381	363	410	377	377
MCS4	574	572	573	376	381	380	–	–	–	553	554	553	MCS3	680	662	655	658	685	659	699	620	641	699	699	699
MCS5	346	343	350	357	376	355	376	344	344	366	359	357	MCS4	406	418	421	412	409	424	466	463	462	429	432	434
MCS6	–	–	–	564	564	515	388	400	433	463	454	446	MCS5	688	684	687	699	687	690	616	690	688	690	690	689
MCS7	500	545	545	477	475	474	554	556	559	349	372	388	MCS6	316	320	329	316	321	324	307	303	307	305	319	313
MCS8	582	527	528	552	552	500	–	–	–	630	629	627	MCS7	585	597	599	424	400	400	–	–	–	678	607	608

*VS, vegetative state; MCS, minimally conscious state; TO, Tone-SON; DO, SDN-SON; SON, subject’s own name; SDN, subject’s derived name; tDCS, transcranial direct current stimulation*.

**Table 5:** Presence or absence of P300 in VS and MCS groups in the TO and DO paradigms at baseline.

Real stimulation	TO paradigm			DO paradigm			Sham stimulation	TO paradigm			DO paradigm		
	Presence	Absence	Total	Presence	Absence	Total		Presence	Absence	Total	Presence	Absence	Total
VS	1	4	5	0	5	5	VS	2	4	6	1	5	6
MCS	7	1	8	6	2	8	MCS	7	0	7	6	1	7

Total	8	5	13	6	7	13	Total	9	4	13	7	6	13

*VS, vegetative state; MCS, minimally conscious state; TO, Tone-SON; DO, SDN-SON; SON, subject’s own name; SDN, subject’s derived name*.

## Discussion

Previous studies have reported the effects of a single session and multiple sessions of tDCS on clinical improvement of patients with DOCs (8, 9, 24), but this therapy is still far from becoming an established clinical practice. There is a pressing need to develop assessment methods to evaluate the treatment effects of tDCS in patients with DOCs, and these methods could also contribute to the understanding of the underlying mechanisms of tDCS. ERPs, mainly the P300 component, have been widely used to detect the electrophysiological correlates of cognitive capabilities, potentially reflecting residual levels of awareness in patients with DOCs (25, 26). In our study, the Fisher’s exact test results demonstrate that the presence of P300 in response to the TO and DO paradigms could be used as a distinctive marker between VS and MCS patients at baseline. The results support the idea that the P300 wave in the oddball paradigms can be used to accurately characterize the level of cognitive preservation in patients with DOCs. However, the present ERP results were based on the analysis of group level rather than an individual participant level. Therefore, an integrated assessment of individual patient with DOCs using electrophysiological methods along with behavioral observation could refine the diagnostic and prognostic evaluation more accurately.

Probing for covert cognitive resources in patients with DOCs using electrophysiological methods requires special stimuli with an established probability of eliciting P300 responses. As demonstrated in the “cocktail party” phenomenon, SON has been frequently used to capture attention and evoke a powerful emotional reaction (27). Our study contrasted SON with SDN and recorded an auditory ERP pattern associated with increasing task complexity (15, 20, 21). The grand average results revealed a larger P300 in response to SON after tDCS compared to before tDCS in MCS patients in the real stimulation group but not in VS patients. This confirms earlier findings that MCS patients have more residual cognitive resources than VS patients.

In our study, we used “passive” paradigms that are independent of the patient’s collaboration and have been shown to have significantly fewer limitations than “active” paradigms, e.g., misunderstanding of task instructions by the patients. The ultimate goal when establishing an ERP task for patients with severe brain injury is not only to elicit cognitively mediated responses but also to not exceed the cognitive capacity of the patients. We believe that actively listening to pitch or counting target stimuli requires higher cognitive capacities compared to just listening. In addition, active ERP tasks require the patients not only to stay awake during the recording but also to understand the commands, be able to hold perceptual representations in their working memory, and complete the task (17). Although it was reported that passive oddball paradigms using the SON were known to elicit responses even during sleep (28), it could also help to detect changes in brain activity due to treatment, which contributed to understand the potential electrophysiological mechanism of tDCS.

The DLPFC plays a central integrative role in motor control and behavior, and it is an important component of the decision-making network (29–31). Studies have showed that as complexity or integration demands during action control increase, the DLPFC is increasingly associated with “top-down” cognitive control (3, 32, 33). The P300 amplitude depends not only on the stimulus saliency but also on the participant’s attentiveness (34, 35). Previous studies have reported that there is a variation in the P300 amplitude according to the amount of focal attention on discriminate stimuli. It can be speculated that the observed tDCS-related consciousness improvements (as assessed by changes in the CRS-R score) are potentially related to improvements in attention resource allocation (as reflected by the P300 amplitude). In contrast, a lack of P300 amplitude modulation might reflect an impairment of processing requiring higher attention. It has been reported that P300 latency can be used as an objective index of stimulus evaluation time and it is sensitive to task complexity. Therefore, a more complex stimulus can lead to a delay in orientation and engagement with new stimuli (16). In our current study, the P300 latency tended to increase with enhancement of stimulus complexity in the MCS group, but there was no significant difference at baseline. A study by Cavinato et al. showed that, in MCS patients, P300 latency was modulated at different levels of stimulus complexity (16). The inconsistency between our results and those of the study by Cavinato et al. might be due in part to the small sample size and the clinical heterogeneity of the patients with DOCs in our study.

Our study showed that there was a statistically significant improvement in the clinical condition of the MCS patients, but not in the VS patients in the real stimulation group. These findings are in line with those of a previous study that demonstrated that out of three patients in prolonged MCSs, all showed mild behavioral improvements after five daily stimulation sessions, but none of the seven VS patients showed behavioral improvements at the end of the stimulation protocol (8). In another recent study of patients with prolonged DOCs (22), repeated tDCS did not lead to a remarkable short-term clinical improvement or to EEG effects. The major difference between the two results may be related to the participants’ postinjury durations. A study demonstrated that consciousness improvement is possible in patients with DOCs despite several months in VS or MCS, suggesting that there remains potential for behavioral improvement even for severely affected patients with DOCs (36). Therefore, we did not exclude the VS or MCS patients for greater than 12 months. In addition, although pediatric brain recovery may differ from adult brain recovery, we did not specially focus on the relevance of ages in patients because of the relatively small sample size in our current study. Statistical analysis showed that there was no statistical difference between the group ages. However, the effect of injury duration and age will be the focus of our following studies.

Recently, an increasing number of studies have explored the effects of non-invasive brain stimulation, including TMS and tDCS, on improvements in consciousness in patients with DOCs (8–10, 37–39). However, in practice, tDCS is easier to use, requires a smaller device, and is less expensive (40) than TMS. In particular, tDCS could be used early in DOC rehabilitation programs due to its safety. The evaluation tools used to assess therapeutic efficacy in patients with DOCs include neurophysiological and functional neuroimaging techniques. However, despite the unquestionable value of magnetoencephalogram, positron emission tomography, and fMRI studies, these procedures cannot be performed at the patient’s bedside. In such cases, the use of electrophysiological recordings is more practical and appropriate.

Although the results are exciting, some questions remain to be addressed in future studies. First, the main limitations of this study are the small sample size and lack of long-term follow-up, and we only presented a group analysis but not an evaluation of the individual response to tDCS. Thus, future investigations should optimize the tDCS parameters in an individual analysis in a larger and more representative sample of patients with DOCs involving longitudinal follow-ups. Second, a methodological limitation of our study is the absence of MRI-based mapping to identify the location of the stimulated area. The large structural lesions in patients with severe brain injury may have potential influence on the effect of tDCS (41). Recent studies demonstrated that the response to tDCS in MCS patients might be related to the residual metabolic activity in brain areas including the left DLPFC (42), as well as high connectivity with regions belonging to extrinsic control network (43). Further studies should employ an individualized tDCS protocol based on patient-tailored brain structure. Third, the difference in stimulus intensity in a number of patients (1 mA instead of 2 mA because of the craniotomy) might add to the variability of the small sample in our study. Future studies should deeply investigate whether or how such change in stimulus intensity had any effect. Fourth, since neurophysiological and functional neuroimaging techniques explore different aspects and consequently provide different information, these methods should be applied together in future studies to provide a broader and more holistic evaluation of therapeutic efficacy in patients with DOCs.

## Conclusion

To the best of our knowledge, this is the first study to provide behavioral and ERP evidence of the effect of tDCS in patients with DOCs. In summary, our study revealed that repeated anodal tDCS of the left DLPFC may produce clinically significant improvements and modulate the P300 amplitude in MCS patients. The study also highlighted the need to associate behavioral evaluation results with neurophysiological results in order to achieve a more objective and accurate assessment of patients with DOCs. Our results demonstrate that ERP recording might serve as an alternative assessment tool for evaluating the effectiveness of DOC treatments and supported the use of tDCS and ERP recording in clinical practice.

## Ethics Statement

Written informed consents were acquired from all the patients’ families or caregivers. The study was conducted according to the Declaration of Helsinki, and the ethics approval was provided by the ethics committee of the hospital.

## Author Contributions

Full access to all the data in the study and responsibility for the integrity of the data and the accuracy of the data analysis: YZ. Study concept and design; acquisition, analysis, or interpretation of data: YZ. Drafting of the manuscript: YZ. Critical revision of the manuscript for important intellectual content: all authors. Statistical analysis: YZ. Obtained funding: WS. Administrative, technical, or material support: GS and RL. Study supervision: JD, SH, and WS.

## Conflict of Interest Statement

The authors declare that the research was conducted in the absence of any commercial or financial relationships that could be construed as a potential conflict of interest.

## Supplementary Material

Figure S1 Grand averages of P300 waves in 16 healthy subjects at Fz, Cz, and Pz in the TO and DO paradigms. The thick line represents the deviant stimuli (subject’s own name).

## Abbreviations

tDCS, transcranial direct current stimulation; DOCs, disorders of consciousness; ERP, event-related potential; VS, vegetative state; MCS, minimally conscious state; CRS-R, Coma Recovery Scale-Revised; DLPFC, dorsolateral prefrontal cortex; SON, subject’s own name; SDN, subject’s derived name; EOG, electrooculography.
