# Supplementary figures and images for: Transcranial Direct Current Stimulation in Patients with Prolonged Disorders of Consciousness: Combined Behavioral and Event-Related Potential Evidence
Source: Front Neurol. 2017 Nov 21;8:620. doi: 10.3389/fneur.2017.00620 (PMC5702306; doi:10.3389/fneur.2017.00620)

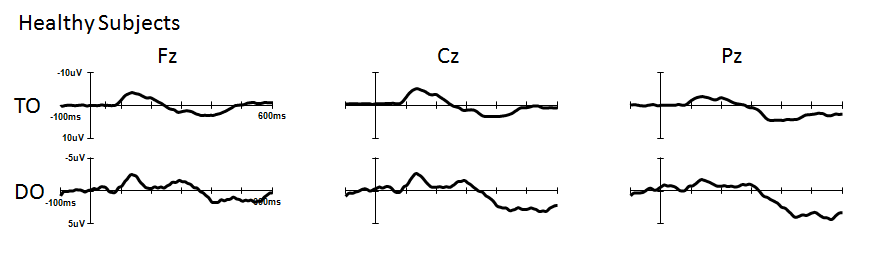

Supplement: Figure S1 — Grand averages of P300 waves in 16 healthy subjects at Fz, Cz, and Pz in the TO and DO paradigms. The thick line represents the deviant stimuli (subject’s own name). [file image_1.tif]
